# Supplementary material for: Sociability is a Multidimensional Trait in Drosophila melanogaster
Source: Behav Genet. 2026 Apr 3;56(2):49–65. doi: 10.1007/s10519-026-10261-5 (PMC13132903; doi:10.1007/s10519-026-10261-5)
Supplement: Supplementary file 1 — Supplementary material 1 (DOCX 1028.3 kb) [file 10519_2026_10261_MOESM1_ESM.docx]

**Supplementary Figures**


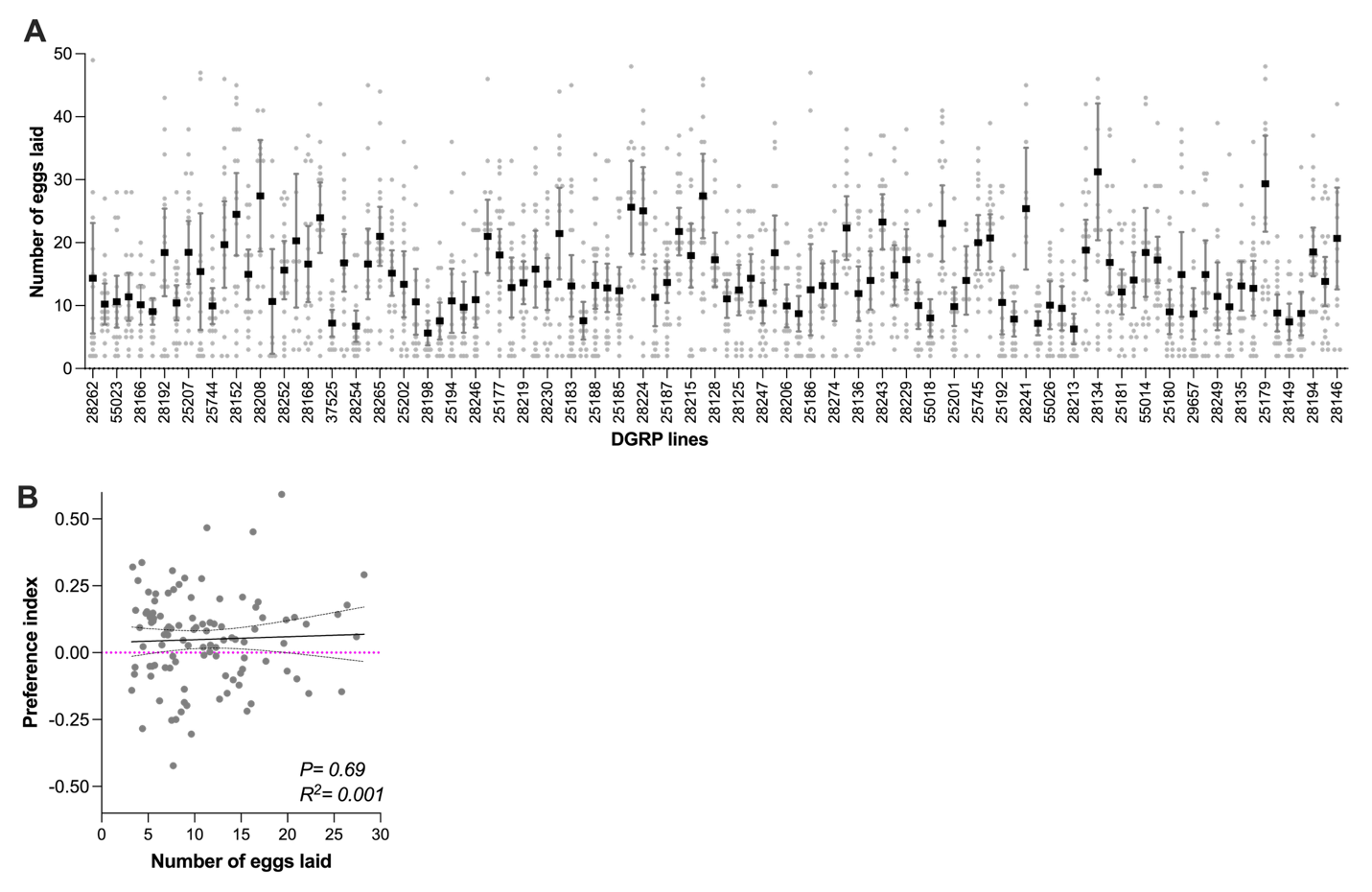


**Figure S1: No correlation between communal egg-laying preference and fecundity between DGRP lines. (A)** Average number of eggs laid in the communal egg-laying preference assay in DGRP lines ordered as in Figure 1B. Replicates ranged from 15 to 29 per line. Error bars indicate 95% confidence interval. (**B**) No correlation between average communal egg-laying preference and number of egg-laid in DGRP lines. For full statistical analysis and methods, see **Supplementary Table S2.**


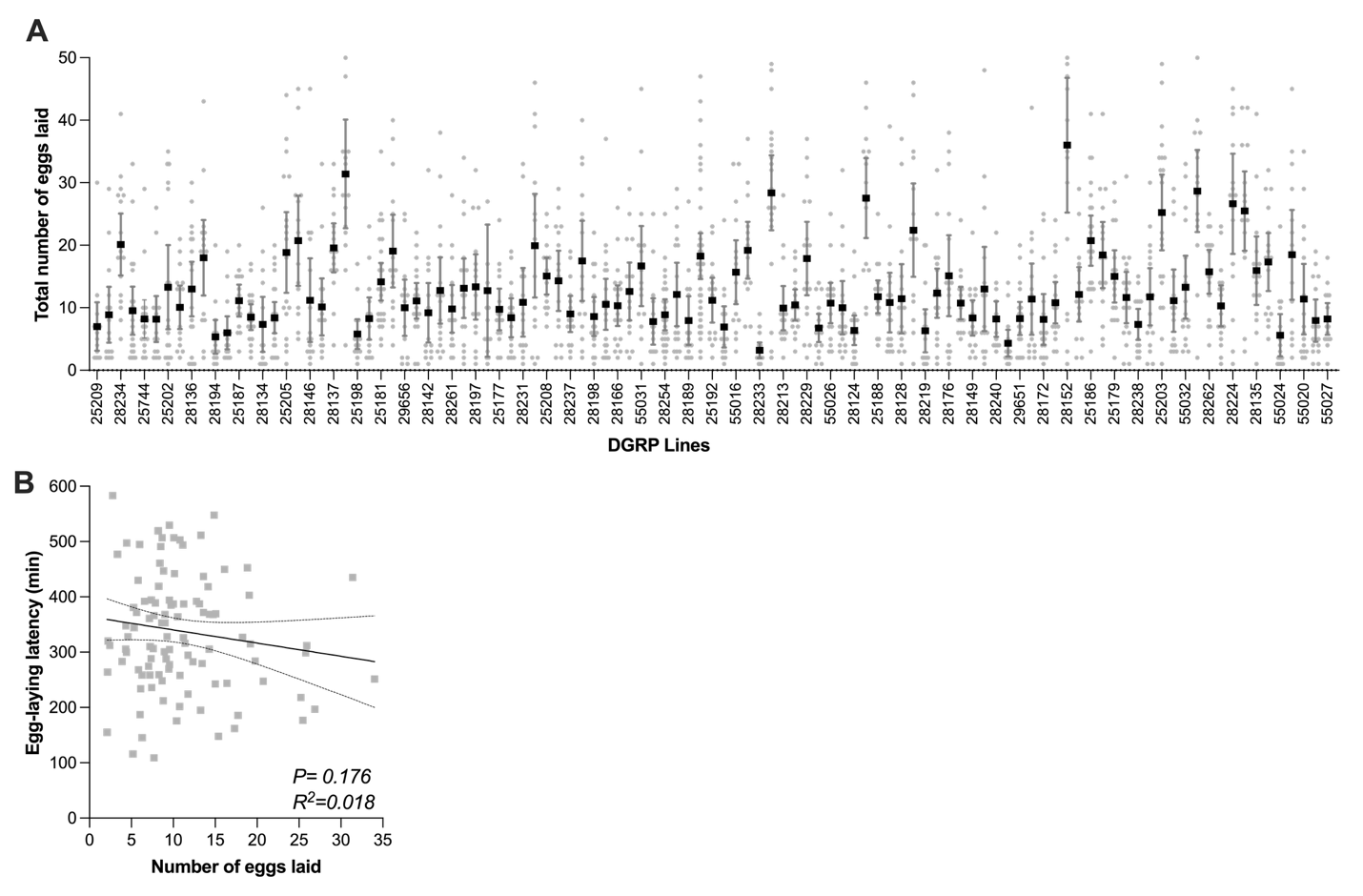


**Figure S2: Lack of correlation between egg-laying latency in group and fecundity between DGRP lines. (A)** Average number of eggs laid in the communal egg-laying assay in DGRP lines ordered as in Figure 2B. Replicates ranged from 15 to 29 per line. Error bars indicate 95% confidence interval. (**B**) No correlation between average egg-laying latency in group and number of egg-laid in DGRP lines. For full statistical analysis, see **Supplementary Table S2.**


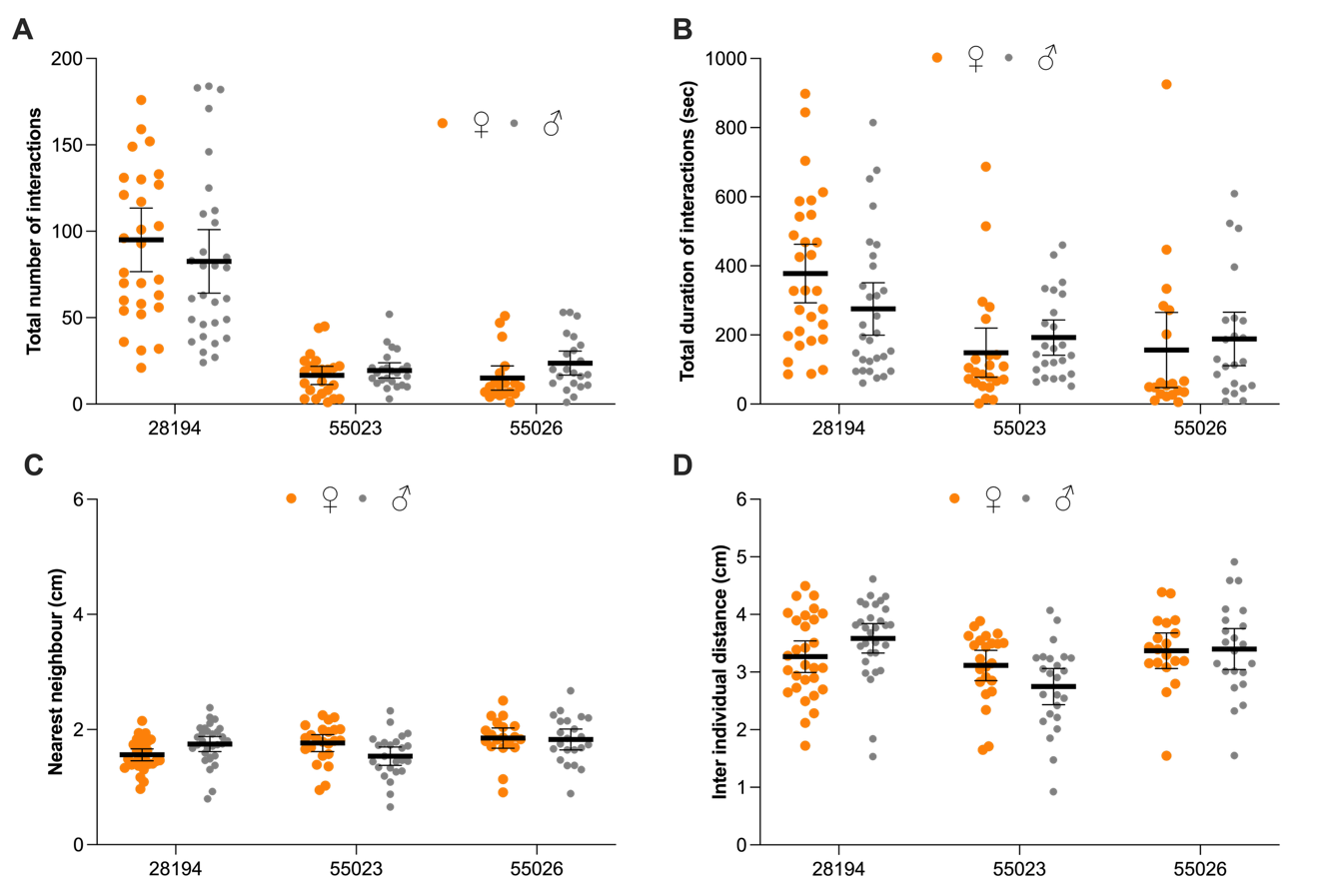


**Figure S3: No significant sexual dimorphism in spontaneous social interaction metrics in three DGRP strains. (A)**Total number, **(B)** duration of all social interactions, **(C)** Average nearest neighbour distance and **(D)** Average inter individual distance between 4 female (orange dots) or male (grey dots) flies from the same DGRP line. For each of the three DGRP lines, between 9 and 27 replicate assays were conducted. Error bars indicate 95% confidence interval. No statistically significant differences were found between males and females in any of the metrics. For full statistical analysis and methods, see **Table S2**.

**Table S1: Drosophila Genetic reference panels DGRP lines used in this study**

| **DGRP lines** | | |
| --- | --- | --- |
| 25174 | 28179 | 55023 |
| 25175 | 28188 | 55024 |
| 25177 | 28189 | 55026 |
| 25179 | 28192 | 55027 |
| 25180 | 28194 | 55031 |
| 25181 | 28197 | 55032 |
| 25182 | 28198 | 83728 |
| 25183 | 28205 |  |
| 25185 | 28206 |  |
| 25186 | 28207 |  |
| 25187 | 28208 |  |
| 25188 | 28213 |  |
| 25189 | 28215 |  |
| 25190 | 28219 |  |
| 25192 | 28220 |  |
| 25193 | 28224 |  |
| 25194 | 28229 |  |
| 25195 | 28230 |  |
| 25198 | 28231 |  |
| 25200 | 28233 |  |
| 25201 | 28234 |  |
| 25202 | 28235 |  |
| 25203 | 28237 |  |
| 25205 | 28238 |  |
| 25206 | 28240 |  |
| 25207 | 28241 |  |
| 25208 | 28243 |  |
| 25209 | 28246 |  |
| 25744 | 28247 |  |
| 25745 | 28249 |  |
| 28124 | 28252 |  |
| 28125 | 28254 |  |
| 28128 | 28256 |  |
| 28132 | 28257 |  |
| 28134 | 28260 |  |
| 28135 | 28261 |  |
| 28136 | 28262 |  |
| 28137 | 28265 |  |
| 28140 | 28274 |  |
| 28142 | 28276 |  |
| 28146 | 29651 |  |
| 28149 | 29656 |  |
| 28152 | 29657 |  |
| 28157 | 37525 |  |
| 28166 | 55014 |  |
| 28168 | 55016 |  |
| 28172 | 55018 |  |
| 28173 | 55019 |  |
| 28176 | 55020 |  |

**Supplementary Table S2**: Summary of statistical analyses organized per figure panels.

| **Fig** | **Test** | **Response variable** | **Explanatory factor** | **Results** | **Post-hoc tests** |
| --- | --- | --- | --- | --- | --- |
| 1B | Quasi-binomial GLMM | Communal egg-laying preference index | DGRP lines | χ² = 2114.7, df = 104, p < 0.001 | NA |
| S1A | Quasi-Poisson GLMM | Total number of eggs | DGRP lines | χ² = 513.65, df = 104, p < 0.001 | NA |
| S1B | Linear regression | Communal egg-laying preference index | Number of eggs laid | slope = 0.001±0.003 SE, F(1, 103) = 0.155, p = 0.694, R² = 0.001 | NA |
| 2B | Gamma GLMM | Egg-laying latency (mins) | DGRP lines | χ² = 352.41, df = 104, p < 0.001 | NA |
| 2C | Binomial GLMM | % first egg laid in light or dark phase | DGRP lines | χ² = 237.56, df = 104, p < 0.001 | NA |
| 2D | Linear regression | Egg-laying latency (mins) | Percentage of first egg during dark phase | slope = 5.41 ± 0.28 SE, F(1,103) = 367.5, p < 0.0001, R² = 0.78 | NA |
| 2E | Type II Wald chisquare test | Egg-laying latency (mins) | Group [isolated, grouped] | χ² = 25.19, df = 1, p < 0.001 | NA |
|  | Type II Wald chisquare test | Egg-laying latency (mins) | DGRP line | χ² = 97.21, df = 29, p < 0.001 | NA |
|  | Type 3 two-way Anova | Egg-laying latency (mins) | Genotype [DGRP line] x Social context [isolated, grouped] | F(29,10) = 3.10, p < 0.001 | 25179: p = 0.017, 25186: p < 0.001, 25203: p = 0.016 |
| S2A | Quasi-Poisson GLMM | Total number of eggs | DGRP lines | χ² = 614.71, df = 104, p < 0.001 | NA |
| S2B | Linear regression | Egg-laying latency (mins) | Number of eggs laid | slope = –2.38 ± 1.75 SE, F(1,103) = 1.86, p = 0.176, R² = 0.018 | NA |
| 3B | Negative binomial GLMM | Frequency of social interactions | DGRP lines | χ² = 781.35, df = 104, p < 0.001 | NA |
| 3C | Tweedie GLMM | Duration of social interactions (sec) | DGRP lines | χ² = 1098.1, df = 104, p < 0.001 | NA |
| 3D | Tweedie GLMM | Inter-individual distance (cm) | DGRP lines | χ² = 549.71, df = 104, *p* < 0.001 | NA |
| 3E | Tweedie GLMM | Nearest-neighbour distance (cm) | DGRP lines | χ² = 575.58, df = 104, *p* < 0.001 | NA |
| S3A | Mann-Whitney with FDR | Total number of interactions | DGRP lines | 28194: 0.41  55023: 0.41  55026: 0.07 | NA |
| S3B | Mann-Whitney with FDR | Total duration of interactions (sec) | DGRP lines | 28194: 0.069  55023: 0.069  55026: 0.219 | NA |
| S3C | Mann-Whitney with FDR | Nearest neighbour distance (cm) | DGRP lines | 28194: 0.026  55023: 0.026  55026: 0.638 | NA |
| S3D | Mann-Whitney with FDR | Inter-individual distance (cm) | DGRP lines | 28194: 0.113  55023: 0.113  55026: 0.937 | NA |
| 4A | Linear regression | Total duration of interactions | Number of interactions | slope = 2.92 ± 0.6 SE, F(1,103) = 23.6, p < 0.0001, R² = 0.19 | NA |
| 4B | Linear regression | Nearest neighbour distance (cm) | Inter individual distance (cm) | slope = 0.52 ± 0.015 SE, F(1,103) = 1068, p < 0.0001, R² = 0.91 | NA |
| 4C | Linear regression | Nearest neighbour distance (cm) | Number of interactions | slope = -0.001 ± 0.00075 SE, F(1,103) = 3.23, p = 0.075, R² = 0.03 | NA |
| 4D | Linear regression | Inter-individual distance (cm) | Number of interactions | slope = 3,306e-005 ± 0.0014 SE, F(1,103) = 0.0006, p = 0.98, R² < 0.01 | NA |
| 4E | Linear regression | Nearest neighbour distance (cm) | Duration of interactions (sec) | slope = -0.0006865 ± 8.974e-005 SE, F(1,103) = 58.52, p < 0.0001, R² = 0.36 | NA |
| 4F | Linear regression | Inter individual distance (cm) | Duration of interactions (sec) | slope = -0.0009172 ± 0.0001 SE, F(1,103) = 24.4, p < 0.0001, R² = 0.19 | NA |
| 5A | Tweedie GLMM | Speed (cm/sec) | DGRP lines | χ² = 1535.1, df = 104, *p* < 0.001 | NA |
| 5B | Tweedie GLMM | Time spent in centre | DGRP lines | χ² = 901.49, df = 104, *p* < 0.001 | NA |
| 5C | Linear regression | Number of interactions | Speed (cm/sec) | slope = -7.803 ± 5.246 SE, F(1,103) = 2.212, p = 0.14, R² = 0.02 | NA |
| 5D | Linear regression | Duration of interactions (sec) | Speed (cm/sec) | slope = -222.3 ± 28.45 SE, F(1,103) = 61.03, p < 0.0001, R² = 0.37 | NA |
| 5E | Linear regression | Inter-individual distance (cm) | Speed (cm/sec) | slope = 0.3266 ± 0.06 SE, F(1,103) = 23.04, p < 0.0001, R² = 0.18 | NA |
| 5F | Linear regression | Nearest neighbour distance (cm) | Speed (cm/sec) | slope = 0.1827 ± 0.04 SE, F(1,103) = 24.67, p < 0.0001, R² = 0.19 | NA |
| 5G | Linear regression | Number of interactions | Time spent in centre (sec) | slope = -0.1312 ± 0.03 SE, F(1,103) = 16.17, p = 0.0001, R² = 0.14 | NA |
| 5H | Linear regression | Duration of interactions (sec) | Time spent in centre (sec) | slope = -0.4703 ± 0.23 SE, F(1,103) = 4.069, p = 0.0463, R² = 0.04 | NA |
| 5I | Linear regression | Inter-individual distance (cm) | Time spent in centre (sec) | slope = -0.0033 ± 0.0003 SE, F(1,103) = 78.52, p < 0.0001, R² = 0.43 | NA |
| 5J | Linear regression | Nearest neighbour distance (cm) | Time spent in centre (sec) | slope = -0.0001 ± 0.0002 SE, F(1,103) = 36.15, p < 0.0001, R² = 0.26 | NA |
| 6A | Linear regression | Communal egg-laying preference index | Egg laying latency (min) | slope = 0.0001 ± 0.0001 SE, F(1,103) = 1.172, p = 0.28, R² = 0.011 | NA |
| 6B | Linear regression | Total number of interactions | Communal egg-laying preference index | slope = 4.337 ± 8.965 SE, F(1,103) = 0.234, p = 0.629, R² = 0.002 | NA |
| 6C | Linear regression | Total number of interactions | Egg laying latency (min) | slope = -0.021 ± 0.013 SE, F(1,103) = 2.424, p = 0.1225, R² = 0.023 | NA |

| **Egg-laying latency** | | |
| --- | --- | --- |
| CHR_SNP | P value | Nearest gene |
| 2R_9595464 | 3,17E-01 | *teiresias* |
| X_16073806 | 5,54E-01 | *-* |
| X_18286749 | 8,93E-01 | *5' nucleotidase B* |
| **Egg-laying site choice** | | |
| CHR_SNP | P value | Nearest gene |
| 2R_19536412 | 2,87E-01 | *retained* |
| 3R_25424357 | 3,72E-01 | *alphabet* |
| 3L_5316719 | 4,15E-01 | *-* |
| 2L_1733959 | 4,18E-01 | *CG17646* |
| 2L_6306511 | 4,38E-01 | *Discoidin domain receptor* |
| 2L_6327068 | 4,38E-01 | *PDZ domain-containing guanine nucleotide exchange factor* |
| 2L_9442210 | 4,38E-01 | *numb* |
| 2L_9447606 | 4,38E-01 | *numb* |
| 2L_9516736 | 4,38E-01 | *CG33298* |
| 2R_6228105 | 4,38E-01 | *slowpoke 2* |
| 2R_7307956 | 4,38E-01 | *Drip* |
| 2R_17333339 | 4,38E-01 | *Syndecan* |
| 3L_4232594 | 4,38E-01 | *Ecdysone-inducible gene L2* |
| 3L_5314283 | 4,38E-01 | *-* |
| 3L_9102510 | 4,38E-01 | *boule* |
| X_10933255 | 4,38E-01 | *vexed* |
| X_19367744 | 4,38E-01 | *-* |
| 2L_18984800_INS | 4,46E-01 | *Swiprosin-1* |
| 2L_19082353_DEL | 4,61E-01 | *Lim3* |
| 3R_23337668 | 4,64E-01 | *-* |
| X_17931182 | 4,91E-01 | *Shaker* |
| X_13227332 | 5,23E-01 | *IncRNA:CR32636* |
| 3R_20804394 | 5,25E-01 | *-* |
| 2L_19365958 | 7,39E-01 | *-* |
| 3L_3349291 | 7,40E-01 | *karst* |
| 3L_7504196 | 7,61E-01 | *CG7546* |
| 3L_5886899 | 8,13E-01 | *-* |
| 3L_3203667 | 9,83E-02 | *gryzun* |
| **Duration of social Interaction** | | |
| CHR_SNP | P value | Nearest gene |
| 2R_19848026 | 1,99E-01 | *alpha-catenin related* |
| 3L_6925887 | 4,43E-01 | *-* |
| 2L_9453441 | 5,36E-01 | *numb* |
| **Nearest Neighbour** | | |
| CHR_SNP | P value | Nearest gene |
| 3R_23568360 | 1,77E-01 | *Ets at 98B* |
| 3R_16368296 | 2,20E-01 | *Signal-transducer and activator of transcription protein at 92E* |
| 3L_22664779 | 2,81E-02 | *Juvenile hormone binding protein 6* |
| 3L_22393694 | 2,99E-01 | *Tenascin major* |
| 3R_16370659 | 3,33E-01 | *Signal-transducer and activator of transcription protein at 92E* |
| X_14638830_INS | 3,34E-01 | *Netrin-B* |
| 3R_18742650 | 3,37E-01 | *klingon* |
| 3L_21063010 | 3,42E-01 | *IQ motif and Sec7 domain ArfGEF* |
| 3L_2184418 | 3,70E-01 | *-* |
| 3L_10080568 | 3,70E-01 | *dpr6 / CG14160* |
| 3L_19684554 | 3,70E-01 | *vermiform* |
| 3R_16367887 | 3,93E-01 | *Signal-transducer and activator of transcription protein at 92E* |
| 3L_5261231 | 7,97E-01 | *alan shepard* |
| 3R_26169493 | 9,97E-01 | *headcase* |

**Table S3:** **Significant Single Nucleotide polymorphisms (SNPs) from GWAS on the indicated phenotypes.** Chromosomal location (CHR) is indicated. When followed by an “INS” indicates a nucleotide insertion and “DEL” indicates a deletion. P-value after Benjamini-Hochberg correction for false discovery rate is indicated, as well as the gene on which this SNP is found.
